# Visceral adipose tissue remodeling in pancreatic ductal adenocarcinoma cachexia: the role of activin A signaling

**DOI:** 10.1038/s41598-022-05660-7

**Published:** 2022-01-31

**Authors:** Pauline C. Xu, Mikyoung You, Seok-Yeong Yu, Yi Luan, Maya Eldani, Thomas C. Caffrey, Paul M. Grandgenett, Kelly A. O’Connell, Surendra K. Shukla, Chandramohan Kattamuri, Michael A. Hollingsworth, Pankaj K. Singh, Thomas B. Thompson, Soonkyu Chung, So-Youn Kim

**Affiliations:** 1 Department of Obstetrics and Gynecology, Olson Center for Women’s Health, College of Medicine, University of Nebraska Medical Center, 985860 Nebraska Medical Center, Omaha, NE 68198 USA; 2 Department of Nutrition, School of Public Health and Health Sciences, University of Massachusetts Amherst, 211 Chenoweth Laboratory, 100 Holdsworth Way, Amherst, MA 01003-9282 USA; 3 Eppley Institute for Research in Cancer and Allied Diseases, University of Nebraska Medical Center, Omaha, NE 68198 USA; 4 Department of Molecular Genetics, Biochemistry, and Microbiology, College of Medicine, University of Cincinnati, Cincinnati, OH 68198 USA

**Keywords:** Cancer metabolism, Gastrointestinal cancer

## Abstract

Pancreatic ductal adenocarcinoma (PDAC) patients display distinct phenotypes of cachexia development, with either adipose tissue loss preceding skeletal muscle wasting or loss of only adipose tissue. Activin A levels were measured in serum and analyzed in tumor specimens of both a cohort of Stage IV PDAC patients and the genetically engineered KPC mouse model. Our data revealed that serum activin A levels were significantly elevated in Stage IV PDAC patients in comparison to age-matched non-cancer patients. Little is known about the role of activin A in adipose tissue wasting in the setting of PDAC cancer cachexia. We established a correlation between elevated activin A and remodeling of visceral adipose tissue. Atrophy and fibrosis of visceral adipose tissue was examined in omental adipose tissue of Stage IV PDAC patients and gonadal adipose tissue of an orthotopic mouse model of PDAC. Remarkably, white visceral adipose tissue from both PDAC patients and mice exhibited decreased adipocyte diameter and increased fibrotic deposition. Strikingly, expression of thermogenic marker UCP1 in visceral adipose tissues of PDAC patients and mice remained unchanged. Thus, we propose that activin A signaling could be relevant to the acceleration of visceral adipose tissue wasting in PDAC-associated cachexia.

## Introduction

Cancer cachexia is a devastating, multifactorial, and often irreversible syndrome that accounts for up to 20% of cancer deaths^1,2^. Unlike starvation where adipose tissue mass is lost while skeletal muscle mass is preserved, cachexia patients lose weight from both compartments^3^. The current international consensus for diagnostic criterion for cancer cachexia is weight loss > 5% over 6 months; or BMI < 20 and any degree of weight loss > 2%; or appendicular skeletal muscle index consistent with sarcopenia and any degree of weight loss > 2%^4^. Pancreatic ductal adenocarcinoma (PDAC) is currently the third leading cause of cancer death in the United States and is projected to become the second leading cause by 2030^5^. Due to lack of early clinical signs, more than 85% of patients present with advanced metastatic disease by time of diagnosis^6^. Prognosis for patients with metastatic disease remains dismal, with a 5 year survival rate of only 2% and median survival time of 3–6 months^7^. A major contributor to the low survival rate is cachexia, a frequent and prominent feature of pancreatic cancer. Notably, 80% of pancreatic cancer patients exhibit symptoms of cachexia at later stages of disease development^8^. Median weight loss close to time of death has been reported to correspond to 25% of pre-illness weight^9^.

Adipose tissue serves as a metabolically dynamic endocrine and energy-storage organ of the body and is primarily composed of adipocytes. In the current research paradigm, most studies and clinical trials have focused on skeletal muscle atrophy rather than adipose tissue loss in cancer cachexia. However, emerging reports indicate that PDAC patients display distinct phenotypes of cachexia development, with either adipose tissue loss preceding skeletal muscle wasting or loss of only adipose tissue^10^. One recent report demonstrates that subcutaneous adipose tissue loss is initiated in patients prior to loss of skeletal muscle and PDAC diagnosis, identifying three distinct and progressive phases of metabolic and soft tissue changes in pre-diagnostic PDAC starting up to 18 months before diagnosis^11^. This phenomenon insinuates that a causal factor for cachexia has already begun to exert systemic effects prior to presentation of prominent cachectic symptoms.

Brown adipocytes may appear after thermogenic stimuli in WAT in a process known as “browning”. A growing body of evidence from animal studies suggests that WAT undergoes “browning” during cancer cachexia, resulting in increased lipid mobilization and energy expenditure^12,13^. This phenotypic switch has been shown to take place at the initial stages preceding skeletal muscle atrophy^14,15^. However, factors that mediate adipose tissue loss in PDAC patients and their contribution to WAT browning in PDAC-mediated cachexia remain to be unveiled.

Activins and inhibins are family members of the TGFβ superfamily involved in a wide range of cellular processes. Activin A is expressed in numerous tissues and plays pivotal roles in regulation of tissue homeostasis, organ development, inflammation, and cell proliferation^16^. The activin signaling pathway has been researched extensively regarding its functions in pancreatic cancer development and progression^17^. Activin signaling can be exploited by cancer cells for their growth advantage paradoxically^18^, despite its evident role in tumor suppression^19^. Multiple studies have implicated a significant role of activin A in skeletal muscle wasting in cancer cachexia and led to propose serum activin A as a prognostic biomarker for cancer patients^20–22^. Surprisingly, few studies have been published regarding activin A’s role in adipose tissue loss in cancer cachexia. One study has been published reporting that the combined administration of activin A and interleukin-6 (IL-6) adenovirus vectors resulted in atrophy of WAT in an animal model^23^, suggesting an interplay between those two factors. A clinical study demonstrated that cachectic patients with gastrointestinal cancer show morphological rearrangement in subcutaneous adipose tissue, resulting in adipose tissue atrophy, formation of fibrotic areas, and immune cell infiltration^24^. Another study that examined the modulation of extracellular matrix (ECM) components in fibrotic subcutaneous adipose tissue obtained from cachectic gastrointestinal cancer patients showed morphological changes in cancer cachexia concomitant with augmented expression of ECM elements through enhanced signaling of the TGFβ pathway^25^. While subcutaneous adipose tissue has traditionally been associated with plasma changes that may function as markers of cancer cachexia^26^, additional studies in pancreatic cancer patients suggested that the rate of visceral adipose tissue loss may be an important indicator of survival. Patients with at least two abdominal CT scans between diagnosis and death, receiving surgery or chemotherapy during cancer progression, were selected. The rate of change (%change/100 days) for subcutaneous adipose tissue was similar to visceral adipose tissue but a change in visceral adipose tissue was significantly correlated with survival in cancer patients^27^.

Here, we investigated the correlation between activin A signaling and visceral adipose tissue remodeling specifically in cachectic PDAC patients, opening a new avenue for finding therapeutic targets to prevent or attenuate the progression of cancer cachexia in patients with pancreatic cancer.

## Materials and methods

All methods were performed in accordance with the relevant guidelines and regulations.

### Patients and sample collection

Human pancreatic specimens and omental adipose tissue from decedents diagnosed with PDAC (*n* = *34*) were obtained through the Rapid Autopsy Program (RAP) for Pancreas in compliance with IRB 091-01. To ensure specimen quality, organs were harvested within three hours postmortem and specimens flash-frozen in liquid nitrogen or fixed in formalin. Informed consent was obtained from all subjects and/or their legal guardian(s). Any studies using tissue solely obtained from the RAP are not considered human subject research because the individuals are not living when the tissue is obtained (per the regulations at 45 CFR 46.102) and therefore would not need IRB approval. The L3 lumbar vertebra landmark was used to estimate fat mass in decedents who underwent CT imaging at time of diagnosis.

Serum samples from age-matched, non-cancer patients with no history of endocrine disorders such as diabetes mellitus (*n* = *25*) were obtained from the Tissue Procurement and Storage Facility though the Biorepository Laboratory of the Nebraska Biobank. Frozen visceral adipose tissue from male non-cancer patients were procured from the Research Tissue Bank (*n* = *7*). The banking of excess discarded de-identified human biological materials obtained solely for clinical purposes does not constitute human subject research subject to 45 CFR 46.

### Animals and orthotopic implantation

This study is reported in accordance with ARRIVE guidelines.

All procedures involving mice were approved by the Institutional Animal Care and Use Committee at UNMC. Animals were provided with food and water ad libitum and kept in Comparative Medicine facilities (UNMC, Omaha, NE). Temperature, humidity, and photoperiod (10/14 photoperiod) were kept constant.

A colony of the transgenic mouse model LSL-Kras^G12D/+^; LSL-Trp53^R172H/+^; Pdx-1-Cre (KPC) was bred by the lab of Dr. Michael A. Hollingsworth as previously described (*n* = 5)^28^. We were granted permission for usage of tumor-derived cell lines KPC8060 and KPC8069 that were generated as previously described^29^. Age-matched healthy C57BL/6 J mice (*n* = 5) were utilized as controls.

A cloned sub-line of human PDAC cell line SUIT-2 (0.5 × 10^6^ S2-013 cells)^30^ was orthotopically implanted into the pancreas of adult male and female athymic nude mice (*n* = 7) by the laboratory of Dr. Pankaj Singh. Age-matched mice who underwent sham operations were utilized as controls (*n* = 7).

### Enzyme-linked immunosorbent assay (ELISA)

Concentrations for activin A were determined using the Activin A ELISA kit (AL-110, Ansh Labs LLC, Webster, TX). Concentrations for interleukin-6 (IL-6) were determined using the Human IL-6 High Sensitivity ELISA kit (BMS213HS, Invitrogen, Carlsbad, CA), and Mouse IL-6 ELISA kit (BMS603-2, Invitrogen). All experiments were performed in duplicates.

### Cell culture and conditioned medium collection

hTERT-HPNE, a normal cell line of intermediary cells formed during acinar-to-ductal metaplasia, was established and granted permission for usage by Dr. Michel M. Ouellette^31^. Human PDAC cell lines BxPC3 (CRL-1687, ATCC, Manassas, VA), PANC-1 (CRL-1469, ATCC), and MIA PaCa-2 (CRL-1420, ATCC); and human cervical adenocarcinoma cell line HeLa (CCL-2, ATCC) were maintained in RPMI-1640 (11875093, Gibco, Waltham, MA) or Dulbecco’s Modified Eagle Medium (DMEM, 12430054, Gibco) with 10% FBS (FBS001-HI, Neuromics Inc., Edina, MN) and 1% penicillin–streptomycin-glutamine (PSG) (10378016, Gibco). An additional 2.5% horse serum (26050070, Gibco) was added to the culture medium of MIA PaCa-2 cells. The KPC8060 and KPC8069 cell lines were maintained in RPMI-1640 with 10% FBS and 1% PSG. All cells were maintained in a 5% CO_2_-humidified atmosphere at 37 °C.

To prepare conditioned medium (CM), fresh culture medium was added to confluent cells and maintained for 24 h prior to collection.

### Ex-vivo culture of adipose tissue

Gonadal and subcutaneous adipose tissue were obtained from four C57BL/6 male mice aged between 9 and 11 weeks and used for the *ex-vivo* culture. Briefly, two gonadal and subcutaneous fat pads from one mouse were divided into four pieces by cutting vertically, and each piece was incubated in either RPMI-1640 (control), CM from KPC8069 cells or CM from KPC8069 cells supplemented with the activin-binding protein follistatin 288 (FST288, 100 ng/mL) for 48 h. The tissues were then fixed in Modified Davidson’s Fixative (64133-50, Electron Microscopy Services, Hatfield, PA) and sectioned for Picro-Sirius Red Staining.

### Real-time reverse-transcription PCR (RT-PCR)

Total RNA was collected using the RNeasy Plus Mini Kit (74134, Qiagen Inc., Germantown, MD) or the RNeasy Lipid Tissue Mini Kit (74804, Qiagen Inc.). One microgram of total RNA from each of the samples were then converted to cDNA using the High-Capacity RNA-to-cDNA™ Kit (4387406, Applied Biosystems, Foster City, CA). TaqMan® probe-based (4304437, Applied Biosystems) or SYBR® Green based (1725121, Bio-Rad Laboratories, Hercules, CA) real-time PCR was performed using primer sequences listed in Supplemental Table S1. The experiment was performed in triplicates using the CFX96™ Real-Time PCR Detection System (185-5096, Bio-Rad Laboratories).

### Immunocytochemistry (ICC), immunohistochemistry (IHC), and immunofluorescence (IF) assays

For ICC, 1 × 10^4^ cells from each of the human pancreatic cancer and normal cell lines were seeded on cover glasses overnight and fixed with 2% formaldehyde. Antigen retrieval was carried out in 10 mM sodium citrate buffer. Samples were then blocked with normal goat serum blocking solution (S-1000-20, Vector Laboratories, Burlingame, CA) and treated with primary antibodies (Supplemental Table S2). Biotinylated or Alexa Fluor™-conjugated secondary antibodies were applied according to primary antibody host species (Supplemental Table S2). Sections for IHC were treated with horseradish peroxidase streptavidin (SA-5004-1, Vector Laboratories) and a DAB substrate kit (34065, ThermoScientific, Waltham, MA) and counter-stained with hematoxylin.

### Adipocyte size measurement

Adipocyte size was measured by analyzing images of hematoxylin–eosin stained adipose tissue sections using Adiposoft 1.14 in Manual Mode as previously described^32^.

### Picro-sirius red staining

To visualize collagen I and III fibers, adipose tissue sections were stained using the Picro-Sirius Red Stain Kit (MER PSR1, Mercedes Scientific, Lakewood Ranch, FL).

### TdT-mediated dUTP nick-end labeling (TUNEL)

TUNEL was performed using the DeadEnd™ Fluorometric TUNEL System (G3250, Promega, Madison, WI).

### Light and fluorescence microscopy

All images were taken with the EVOS M7000 Imaging System (AMF700, Invitrogen, Carlsbad, CA).

### Western blot analysis

Samples were homogenized in RIPA buffer (89900, Thermo Scientific) combined with cOmplete™ Protease Inhibitor Cocktail (CO-RO, Roche Diagnostics USA, Indianapolis, IN) and PhosSTOP™ (PHOSS-RO, Roche Diagnostics USA). Equal amounts of protein were loaded onto a 4–20% polyacrylamide gel (4561094, Bio-Rad Laboratories) and transferred to a nitrocellulose or PVDF membrane. The primary and secondary antibodies used are described in Supplemental Table S2.

### Statistical analysis

Data were presented by mean ± S.E.M. Serum activin A and IL-6 levels were compared between groups using a nonparametric Wilcoxon rank-sum test due to the departure from the normality. Pearson correlation was used to determine the correlation between activin A and IL-6 levels and activin A levels with metastasis sites and body mass index for patients with PDAC. All figures with statistical analyses were generated using PRISM version 9.2.0 (GraphPad Software Inc., San Diego, CA, www.graphpad.com). Values of *p* < 0.05 were considered statistically significant. *p < 0.05; **p < 0.01; ***p < 0.001; ****p < 0.0001; n.s., not significant.

## Results

### Visceral adipose tissue loss can be correlated with increasing systemic activin A levels contributed by pancreatic ductal adenocarcinoma (PDAC) tumors

**Figure 1:**
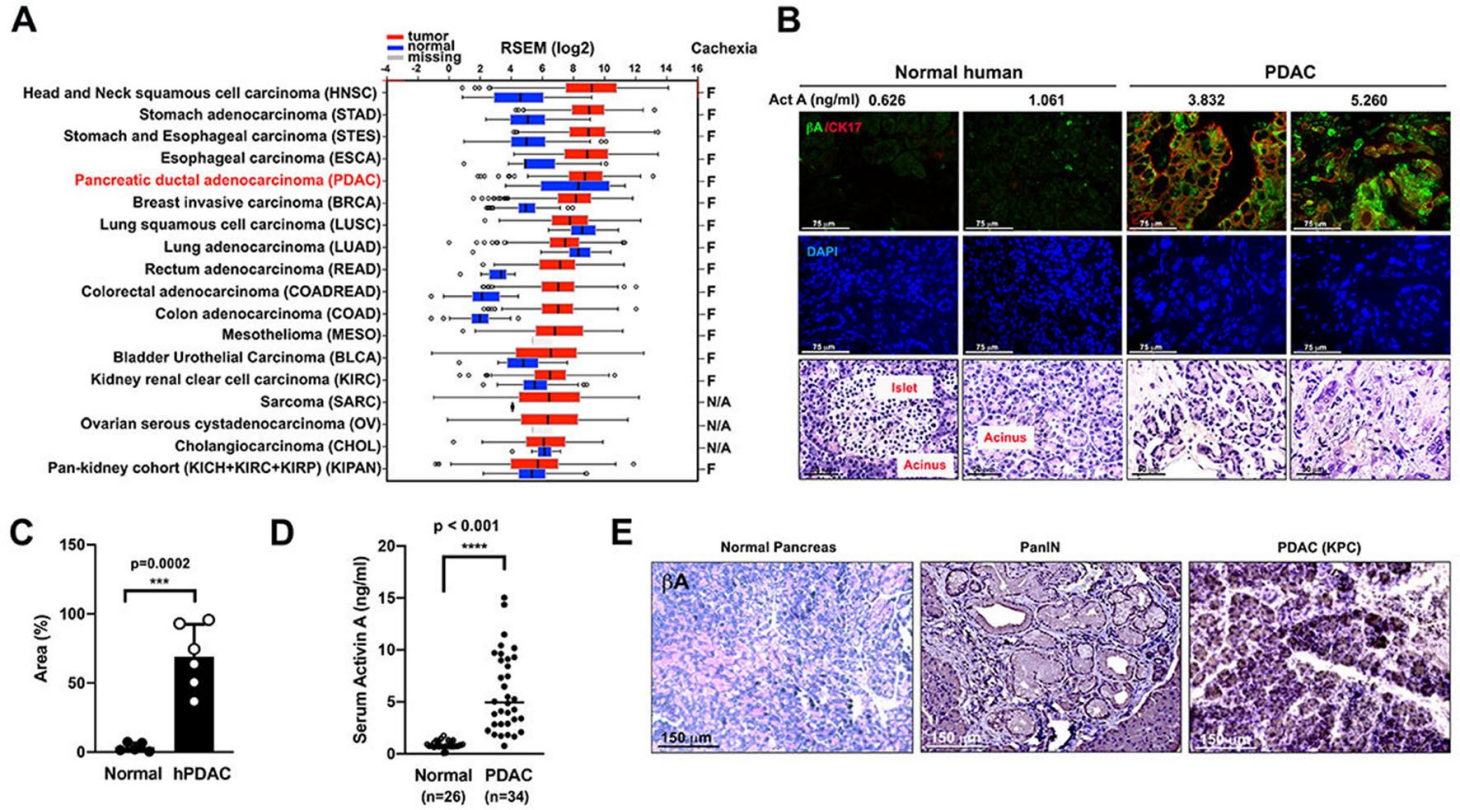
Activin A is highly expressed in tumor specimens from PDAC patients and enters systemic circulation. (**A**) Gene expression analysis of *INHBA* in 18 tumor types of The Cancer Genome Atlas (TCGA) compared with corresponding matched normal (TCGA) tissues using RNA-Seq by Expectation–Maximization (RSEM). Sources for the statistics of cancer cachexia prevalence can be found in Supplemental References. (**B**) Representative immunofluorescence images of activin A (βA, AF488) and keratin 17 (CK17, AF568) expression in non-cancerous pancreatic tissue and PDAC tumor specimens. Nuclei were stained with DAPI. Scale bar = 75 µm. Corresponding serum activin A levels are indicated in ng/ml. Representative H&E images are displayed on the third row. Scale bar = 50 µm. (**C**) Area percentage of green pixels indicating activin A expression in immunofluorescence images from non-cancerous pancreatic tissue (*n* = *5*) and PDAC tumor specimens (*n* = *6*). Each dot represents the average green pixel area percentage per image as measured by Image J. (**D**) Measurement of activin A levels in sera of non-cancer patients (*n* = *25*) and PDAC patients (*n* = *34*). (**E**) Representative immunohistochemical images of activin A expression in pancreas tissue from healthy control as well as PanIN lesions and tumor specimens from KPC mice.

We first analyzed the correlation between *INHBA* (inhibin subunit βA) expression and the prevalence of cancer cachexia in 18 types of cancer using RNA-Seq data from The Cancer Genome Atlas (TCGA). Notably, the expression of *INHBA* was greater in PDAC tumor tissue in comparison to that in matched normal tissue (Fig. 1A).

We next examined the expression of activin A (βA) in PDAC tumor specimens and non-cancerous pancreatic tissues. We also investigated the expression of keratin 17 (CK17), a marker of the most lethal molecular subtype of pancreatic cancer^33^, to evaluate potential correlation with activin A. Both activin A (βA) and CK17 appear to be highly expressed in PDAC tumor tissues in comparison to non-cancerous pancreatic tissues. A large portion of βA-positive cells were found to overlap with CK17 signal (yellow, Supplemental Fig. S1A, B). In contrast to undetectable levels of activin A (βA) in non-cancerous pancreatic tissues, activin A is highly expressed in PDAC tumor tissues (Fig. 1 B, C and Supplemental Fig. S1A, B). Representative histological sections support the development of PDAC in these samples (Fig. 1B and Supplemental Fig. S1C).

To determine whether activin A detected in pancreatic tumor tissue could contrinute to changes in levels of activin A in the circulation, we measured activin A from sera from deceased donors with Stage IV PDAC (*n* = 34) and from healthy donors (*n* = 25). Serum activin A concentrations of PDAC donors had an average of 5.85 ng/mL, while serum activin A concentrations of healthy donors had an average of 0.91 ng/ml (Fig. 1D). The mean serum IL-6 concentration of the same population of PDAC donors was 270.90 pg/mL, and the mean serum IL-6 concentration of healthy donors was 34.93 pg/ml (Supplemental Fig. S1E). No significant correlation could be observed between serum activin A and serum IL-6 concentrations in our cohort of PDAC patients (Supplemental Fig. S1F). No statistically significant gender differences in serum activin A and IL-6 levels could be observed within our PDAC patient population (Supplemental Fig. S1D, G and H). Attempts to correlate serum activin A and body mass index in PDAC patients yielded a Pearson correlation coefficient of − 0.29 with *p* = 0.10, while serum activin A had no correlation with the number of metastasis sites (Supplemental Fig. S1I, J). Interestingly, activin A expression could also be detected in pancreatic intraepithelial neoplasia (PanIN) lesions that are considered precursors to PDAC from KPC mice^34^ (Fig. 1E). Accordingly, activin A expression was detected in the draining lymph node, spleen, liver, and lung of multiple cachectic KPC mice, reflecting a secondary systemic response to activin A from the PDAC tumor (Supplemental Fig. S2C).

**Figure 2:**
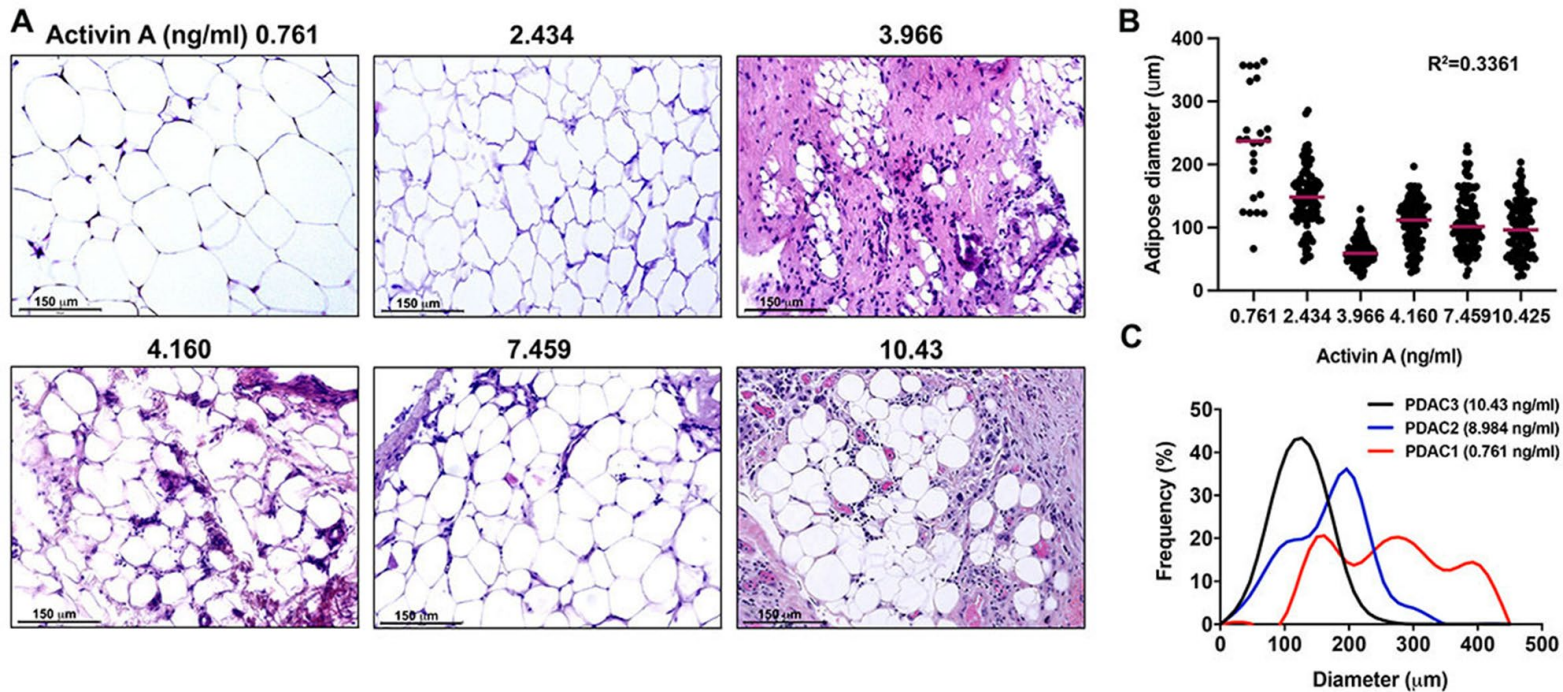
Serum activin A levels can be correlated with loss of visceral adipose tissue in PDAC patients. (**A**) H&E images of visceral adipose tissue from PDAC patients with corresponding activin A levels in ng/ml. Scale bar = 150 µm. (**B**) Measurement of adipocyte diameter in visceral adipose tissue from PDAC patients. The diameters of adipocytes were measured from several histological sections. 6 sections, 0.761 ng/ml; 14 sections, 2.434 ng/ml; 14 sections, 3.966 ng/ml; 11 sections, 4.160 ng/ml; 10 sections, 7.459 ng/ml; 6 sections, 10.425 ng/ml. Corresponding serum activin A levels are included in ng/ml. (**C**) Frequency curve of adipocyte diameter in visceral adipose tissue from patients with low serum activin A (0.761 ng/ml, red line), elevated serum activin A (8.984 ng/ml, blue line), and increasingly elevated serum activin A (10.425 ng/ml, black line). The diameters of adipocytes were measured with 6 histological sections each.

Visceral adipose tissue collected from the omenta of PDAC patients displayed a significant histological changes in correspondence with elevated serum activin A including possible fibrous connective tissue (Fig. 2A). We first observed a decrease in white adipocyte diameter inversely correlating with serum activin A levels (Fig. 2B). We also noted a frequency shift towards smaller adipocytes (Fig. 2C) in patients with higher than average serum activin A levels. We also sought to assess fat mass in PDAC patients by quantifying tissue area in transverse CT-scan images from the third lumbar vertebrae. CT images were matched with serum activin A levels of respective donors, with subcutaneous (blue) and visceral (yellow) fat (Supplemental Fig. S3). While there appears to be decreasing body fat with increasing activin A levels, the activin A levels used were measured after death and the CT images were captured at time of diagnosis.

### Elevated activin A levels observed in patients can be recapitulated in tumor-derived cell lines and an animal model of PDAC

**Figure 3:**
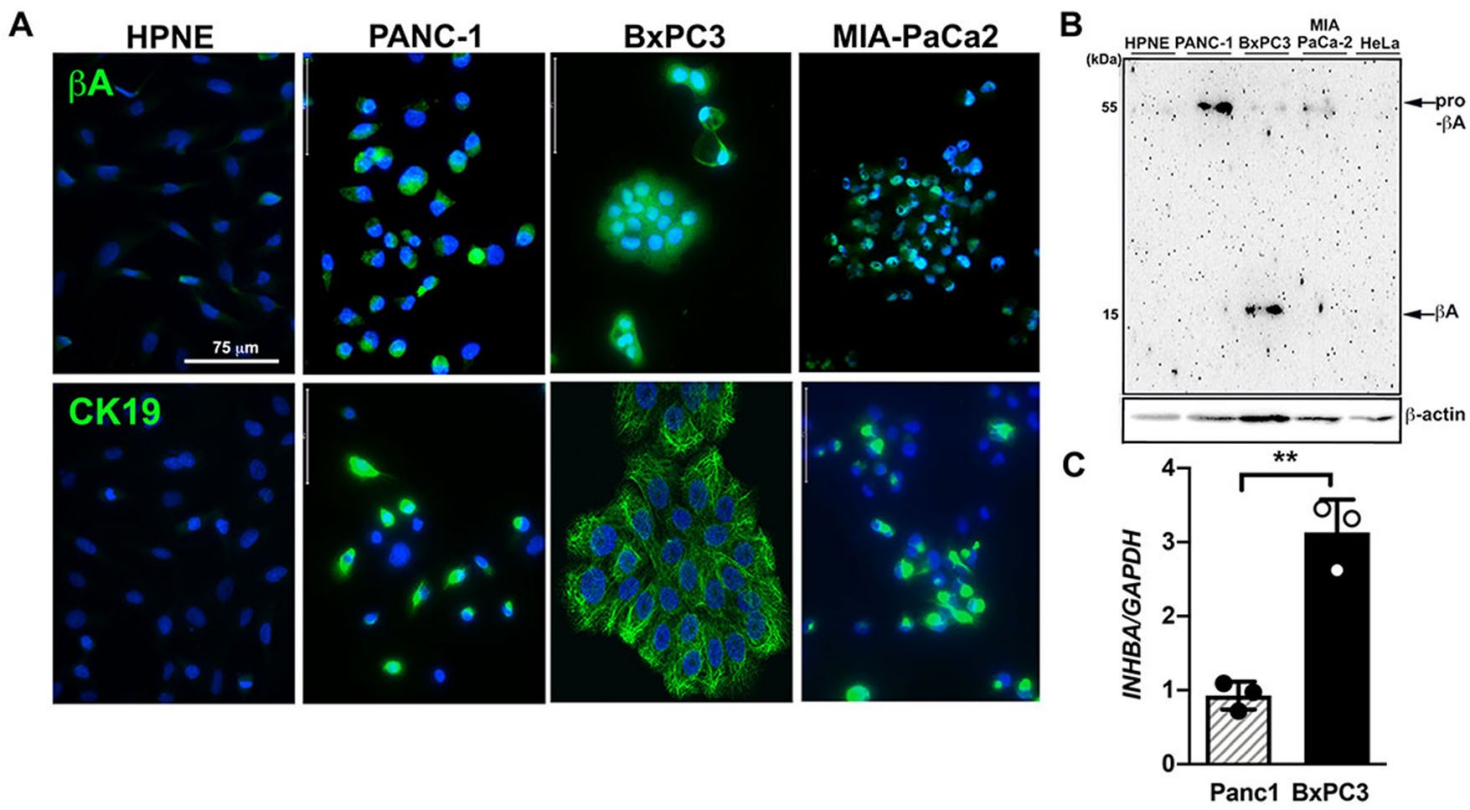
Activin A is highly expressed in tumor-derived cell lines from PDAC patients. (**A**) Immunofluorescence images of activin A (βA, top panels, AF488) and cytokeratin 19 (CK19, bottom panels, AF488) in hTERT-HPNE, PANC-1, BxPC-3 and MIA PaCa-2 cells. Nuclei were stained with DAPI. Scale bar = 75 µm. (**B**) Immunoblotting analysis of activin A expression in hTERT-HPNE, PANC-1, BxPC3, MIA PaCa-2 and HeLa cell lysates. (**C**) Relative *INHBA* mRNA expression levels in PANC-1 and BxPC-3 cells.

BxPC3, PANC-1 and MIA PaCa-2 cells derived from PDAC patients revealed comparable upregulation of activin A expression in contrast to low basal expression in normal pancreatic acinar-to-ductal metaplasia hTERT-HPNE cells (Fig. 3A). Elevated mature activin A could be detected in BxPC3 cell lysates, while PANC-1 and MIA PaCa-2 cell lysates revealed elevated levels of pro-activin A (Fig. 3B). BxPC3 cells possessed a three-fold change in *INHBA* mRNA in comparison to PANC-1 cells (Fig. 3C).

**Figure 4:**
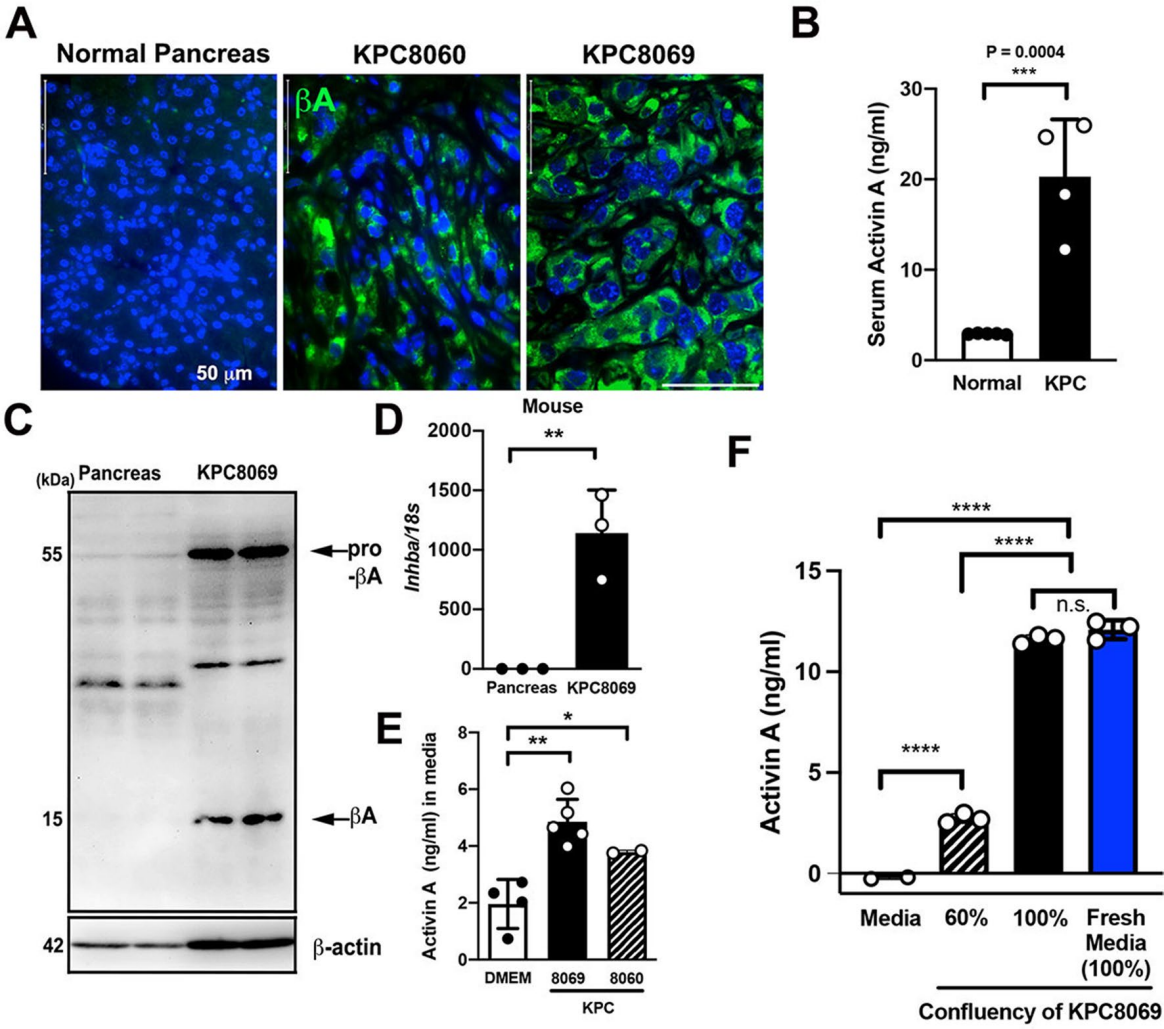
Activin A is highly expressed in tumor specimens and tumor-derived cell lines of genetically engineered mouse models of PDAC. (**A**) Immunofluorescence images of activin A (AF488) in normal mouse pancreas tissue and tumor specimens of KPC 8060 and KPC 8069 mice. Scale bar = 50 µm. (**B**) Measurement of serum activin A levels in healthy control C567BL/6 J and KPC mice. (**C**) Immunoblotting analysis of activin A expression in healthy control C57BL/6 J mouse pancreas tissue and KPC8069 cell lysates. (**D**) Relative *Inhba* mRNA expression levels in healthy control C57BL/6 J mouse pancreas tissue and KPC8069 cells. (**E**) Measurement of activin A concentration in CM from KPC8060 and KCP8069 cells along with RPMI-1640. (**F**) Measurement of activin A concentration in CM from KPC8069 cells at 60% confluency, 100% confluency and 100% confluency following reintroduction (a blue bar) of fresh culture medium.

We also observed high expression of activin A in KPC tumor specimens in comparison to healthy control (Fig. 4A) and a significant elevation in measured circulating activin A levels in the serum of KPC mice (Fig. 4B). In contrast to virtually no pro-activin A and mature activin A detected in normal mouse pancreas tissue, the expression levels of pro-activin A and mature activin A were dramatically elevated in KPC8069 cell lysates (Fig. 4C). KPC8069 cells demonstrated 1000-fold more *Inhba* mRNA than healthy mouse pancreatic tissue (Fig. 4D). To determine whether PDAC cells directly secrete activin A into growth media, we measured activin A levels in conditioned media (CM) from KPC8060 and KPC8069 cells. CM from both KPC8060 and KPC8069 cells showed higher activin A secretion (Fig. 4E). CM from KPC 8069 cells collected at 60% and 100% confluency reflected an increase in activin A secretion dependent on cell density. Interestingly, activin A secretion remained just as high after replacement with fresh culture medium at 100% confluency (Fig. 4F).

### Visceral adipose tissue loss can be correlated with increasing systemic activin A levels contributed by pancreatic ductal adenocarcinoma (PDAC) tumors in an orthotopic mouse model

**Figure 5:**
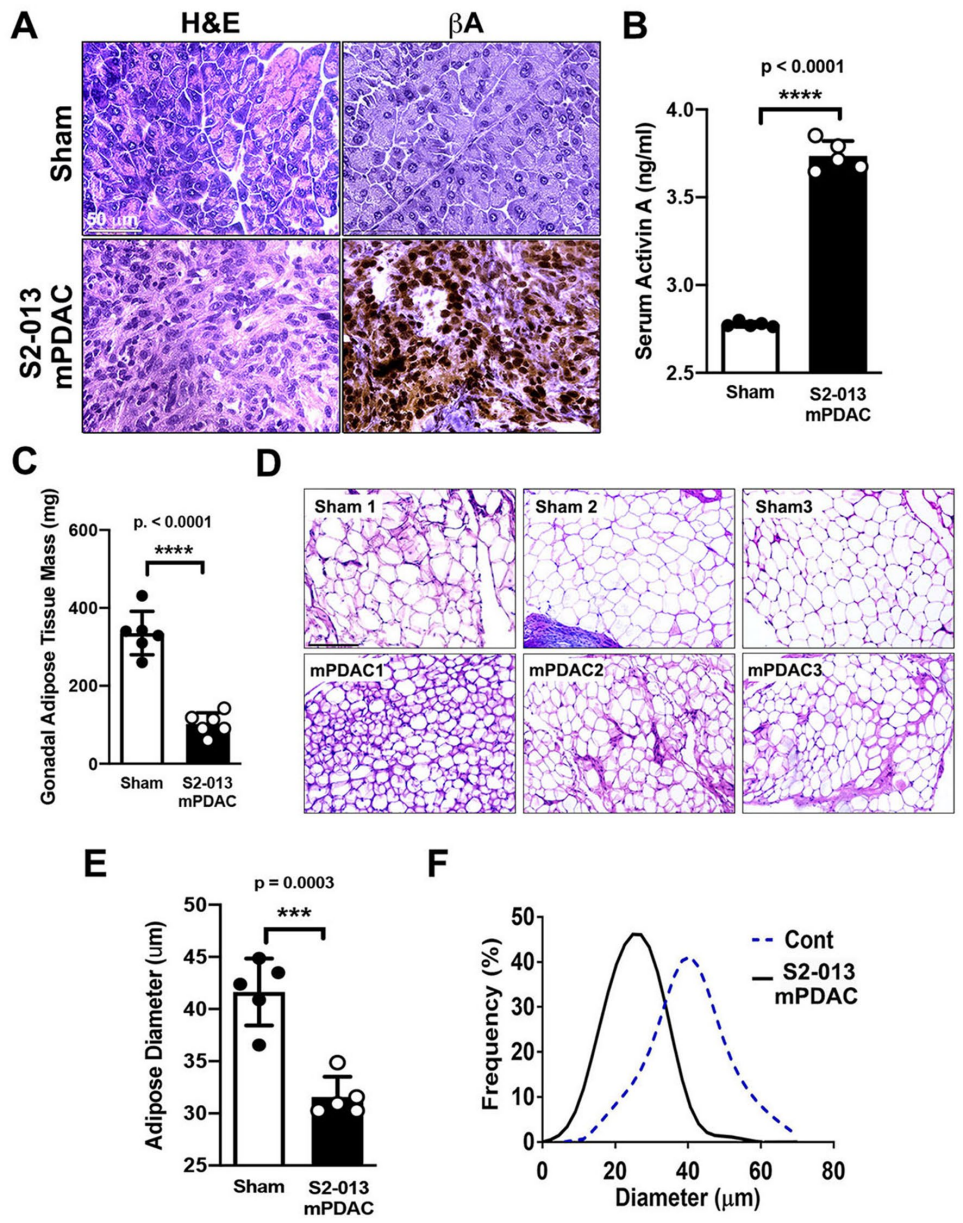
Activin A can be correlated with loss of gonadal visceral adipose tissue in an orthotopic implantation model of PDAC. (**A**) Representative immunohistochemical images of activin A expression in healthy control C57BL/6 J pancreatic tissue and tumor specimens of S2-013 mPDAC. Scale bar = 50 µm. (**B**) Measurement of serum activin A levels in sham and S2-013 mPDAC. (**C**) Mass of gonadal fat pad. (**D**) H&E images of adipose tissue from the gonadal fat pad in sham and S2-013 mPDAC. Scale bar = 150 µm. (**E**) Measurement of adipocyte diameter in gonadal adipose tissue from sham and S2-013 mPDAC. The diameters of adipocytes were measured from several histological sections (*n* = *5*). The average adipocyte diameter from each animal was marked with a dot. F. Frequency curve of adipocyte diameter in gonadal fat pad of sham mice (dashed line) and S2-013 mPDAC (solid line).

We evaluated the expression of activin A in an orthotopic mouse model implanted with S2-013 cells. Tumor tissue sections from S2-013 mPDAC revealed greatly elevated activin A expression in tumors from tumor-bearing mice in comparison to sham (Fig. 5A). Serum levels of activin A also reflected the growth of S2-013-derived tumors (Fig. 5B). However, in accordance with human data, there was no significant correlation between circulating activin A and IL-6 levels (Supplemental Fig. S2A). In addition to reduced body weight, the mass of gonadal fat pads from S2-013 mPDAC (*n* = 7) was greatly reduced (Fig. 5C), with smaller white adipocytes (Fig. 5D) with possible fibrous connective tissue and a shift toward small-diameter adipocyte size in comparison to sham (Fig. 5E, F).

**Figure 6:**
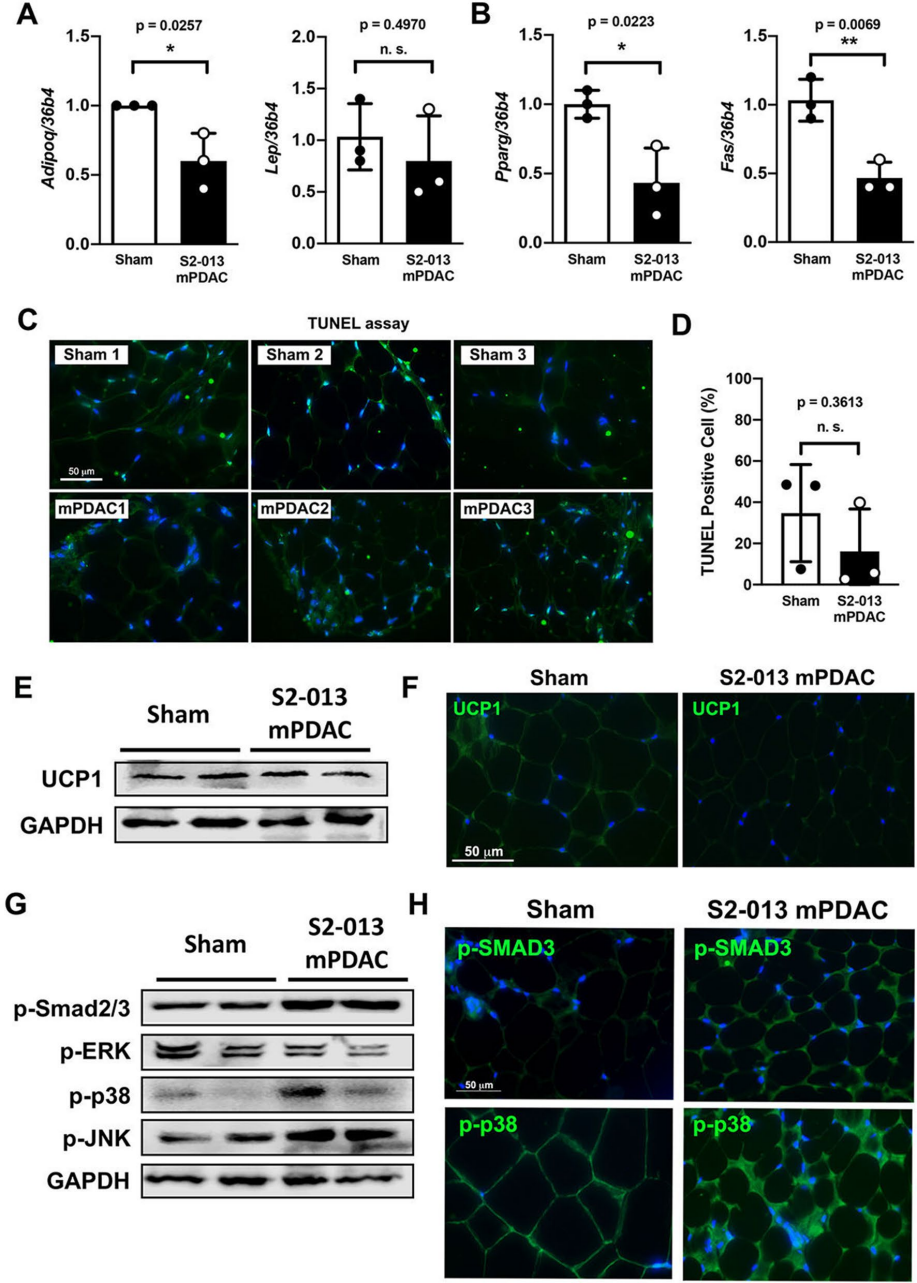
Visceral adipose tissue remodeling in an orthotopic implantation model of PDAC does not rely on increased browning of white adipose tissue. (**A**) Relative mRNA expression levels of *Adipoq* and *Lep* in gonadal adipose tissue from sham and S2-013 mPDAC. (**B**) Relative mRNA expression levels of *Pparg* and *Fas* in gonadal adipose tissue from sham and S2-013 mPDAC. (**C**) TUNEL assay with adipose tissue from the gonadal fat pad of sham (*n* = *3*) and S2-013 mPDAC (*n* = *3*). (**D**) Area percentage of green pixels indicating TUNEL-positive signals as measured by Image J. Each dot represents the percentage of TUNEL-positive signals in the gonadal fat pad of one animal. (**E**) Representative immunofluorescence image of UCP-1 expression in the gonadal fat pad of sham control and S2-013 mPDAC. Nuclei were stained with DAPI. (**F**) Immunoblotting with UCP1 in gonadal adipose tissue from sham and S2-013 mPDAC. (**G**) Immunoblotting with the markers for canonical activin A (p-Smad2/3) and ERK/MAPK pathways in adipose tissue from the gonadal fat pad of sham and S2-013 mPDAC. (**H**) Representative immunofluorescence images of p-SMAD3 and p-p38 expression in adipose tissue from the gonadal fat pad of sham and S2-013 mPDAC. Nuclei were stained with DAPI. Scale bar = 50 µm for all microscope images.

We next investigated the metabolic changes in visceral adipose tissue in response to pancreatic cancer development. Expression level of adiponectin, a marker for insulin sensitivity, was significantly reduced, suggesting insulin-resistance. Paradoxically, there was no significant difference in leptin transcription levels, despite markedly reduced adipocyte size, signifying adipose tissue dysfunction in the cachectic state in S2-013 mPDAC (Fig. 6A). Next, we tested adipocyte-specific gene expression levels. Analyses of S2-013 mPDAC revealed a significant reduction in the expression of *Pparg*, a major transcription factor for adipocyte differentiation, and fatty acid synthetase (*Fas*) (Fig. 6B). To address the role of apoptotic programmed cell death in cachectic fat loss, we labeled DNA fragmentation on sections of adipose tissue from both sham and S2-013 mPDAC. The percentage of TUNEL-positive cells in adipose tissue from S2-013 mPDAC did not increase in comparison to that of sham (Fig. 6C, D). These results suggest that loss of adipose tissue in S2-013 mPDAC is not driven by apoptotic pathways.

### Activin A secreted from PDAC tumor cells contributes to loss of visceral adipose tissue independent of WAT browning and instead contributes to fibrosis of adipose tissue as a mechanism for adipocyte remodeling

Unexpectedly, we were unable to detect an increase in classical brown adipose tissue marker uncoupling protein 1 (UCP1) protein expression per adipocyte in S2-013 mPDAC samples from the gonadal fat pad (Fig. 6E, F). To investigate the downstream signaling cascades potentially involved with adipose tissue remodeling in favor of fibrosis, we analyzed the expression of phosphorylated SMAD2/3 (p-SMAD2/3) and ERK/MAP kinase signaling molecules. In addition to an increase in the expression of p-SMAD2/3 (Fig. 6G) and p-SMAD3 (Fig. 6H) in the adipose tissue of S2-013 mPDAC, we observed an increase in p-p38 and p-JNK expression and a marginal decrease in p-ERK expression, indicating that modulation of MAP kinase signaling pathways could be related with adipose tissue remodeling in favor of fibrosis.

**Figure 7:**
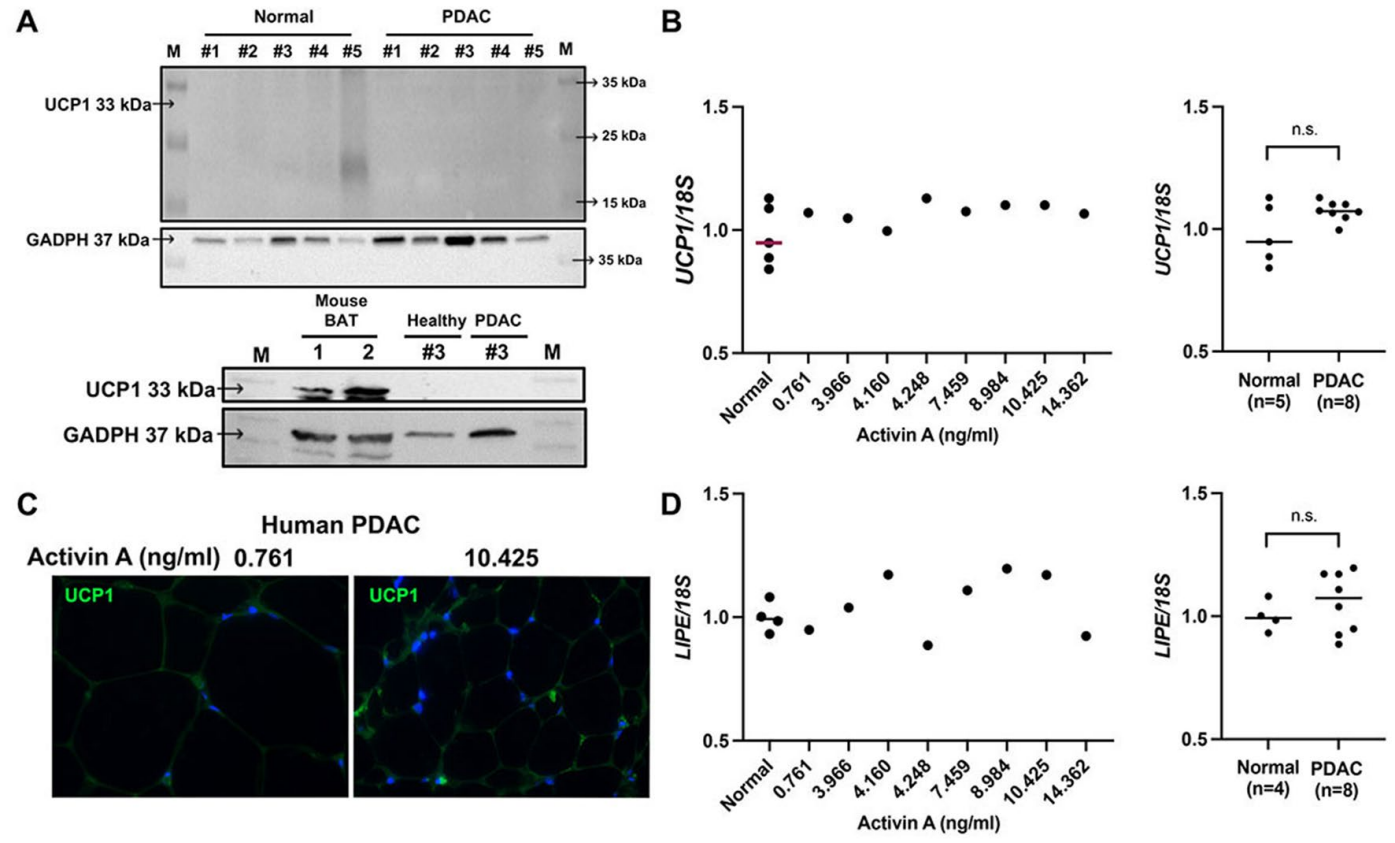
Visceral adipose tissue remodeling in PDAC patients does not rely on increased browning of white adipose tissue. (**A**) Immunoblotting with UCP1 (33 kDa) in visceral adipose tissue of non-cancer patients (*n* = *5*) and PDAC patients (*n* = *5*) (top). Immuoblotting with UCP1 in visceral adipose tissue of a non-cancer patient and a PDAC patient with intrascapular brown adipose tissue from a healthy control mouse as a positive control (*n* = *2*) (bottom). (**B**) Relative mRNA expression levels of *UCP1* in visceral adipose tissue of non-cancer patients (*n* = *5*) and PDAC patients (left). Corresponding serum activin A levels are included in ng/ml. Statistical analysis of relative mRNA expression levels of *UCP1* in visceral adipose tissue of non-cancer patients (*n* = *5*) and PDAC patients (*n* = *8*) (right). n.s., not significant. (**C**) Representative immunofluorescence images of UCP1 expression in visceral adipose tissue from patients with low and high circulating activin A levels included in ng/ml. Nuclei were stained with DAPI. (**D**) Relative mRNA expression levels of *HSL (LIPE) in* visceral adipose tissue of non-cancer patients and PDAC patients (left). Corresponding serum activin A levels are included in ng/ml. Statistical analysis of relative mRNA expression levels of *LIPE* in adipose tissue of non-cancer patients (*n* = *4*) and PDAC patients (*n* = *8*) (right). n.s., not significant. Corresponding serum activin A levels are included in ng/ml.

Since we did not observe adipose tissue browning in the setting of tumor-bearing mice, we next examined if white visceral adipose tissue in PDAC patients underwent a transition towards brown adipose tissue at gene and protein levels. Accordingly, we were unable to detect any increase in UCP1 expression in adipose tissue of Stage IV PDAC patients (Fig. 7A). Consistently, gene expression of *UCP1* in visceral adipose tissue revealed no significant change with increasing circulating activin A levels in PDAC patients and no significant change between PDAC patients and non-cancer patients (Fig. 7B). Accordingly, we were unable to detect an increase in UCP1 protein expression per adipocyte in PDAC patients (Fig. 7C). The gene expression levels of *HSL* measured in visceral adipose tissue from PDAC patients and non-cancer patients again revealed no significant change with increasing circulating activin A levels in PDAC patients and no significant change between patient groups (Fig. 7D).

**Figure 8:**
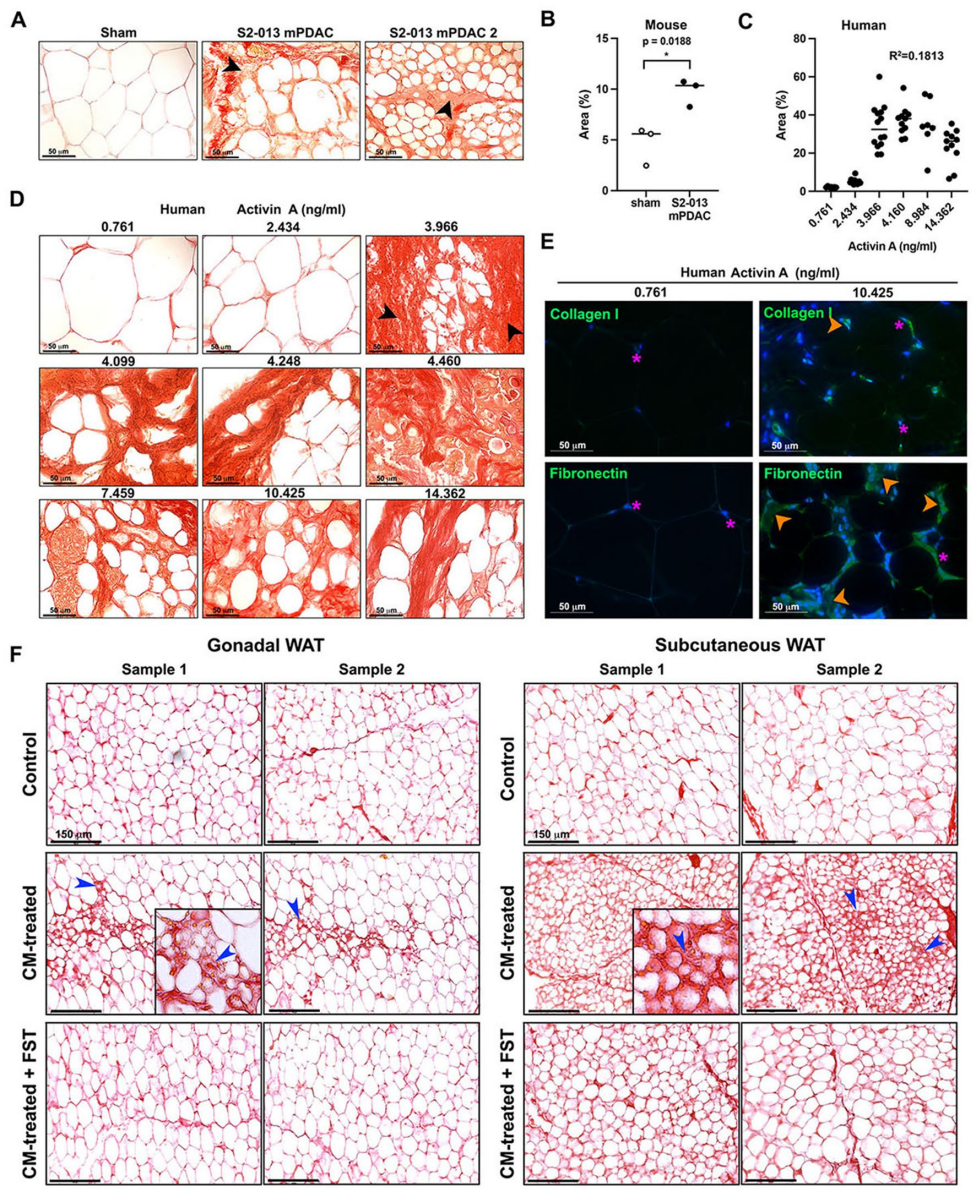
Fibrotic remodeling of visceral adipose tissue in PDAC patients and an orthotopic implantation model appears to correlate with activin A. (**A**) Picro-Sirius Red staining of gonadal adipose tissue of sham control and S2-013 mPDAC. Arrows indicate areas of collagen deposition. (**B**) Picro-Sirius Red staining of visceral adipose tissue in PDAC patients with corresponding activin A levels in ng/ml. Arrows indicate areas of collagen deposition. (**C**) Area percentage of red pixels indicating collagen I/III deposition in gonadal adipose tissue from sham control and S2-013 mPDAC as measured by Image J. The average area percentage of red pixels from each animal was marked with a dot. (**D**) Area percentage of red pixels indicating collagen I/III deposition in visceral adipose tissue from PDAC patients with corresponding activin A levels in ng/ml. The areas of collagen I/III deposition were measured from several histological sections. 7 sections, 0.761 ng/ml; 13 sections, 2.434 ng/ml; 14 sections, 3.966 ng/ml; 11 sections, 4.160 ng/ml; 7 sections, 8.984 ng/ml; 11 sections, 14.362 ng/ml. Each dot indicates the area per section. (**E**) Immunofluorescence images with collagen I and fibronectin in visceral adipose tissue from patients with low and high serum activin A in ng/ml. Nuclei were stained with DAPI. Purple stars indicate adipocyte nuclei, while orange arrows indicate areas of signal. Scale bar = 50 µm for all microscope images. (**F**). Picro-Sirius Red staining of gonadal and subcutaneous white adipose tissue (WAT) obtained from C57BL/6 mice and cultured ex vivo with RPMI1640 (control), CM from KPC8069 cells, or CM from KPC8069 cells supplemented with FST288. Blue arrows indicate areas of collagen deposition.

To confirm ECM deposition in cachectic adipose tissue, we stained adipose tissue from S2-013 mPDAC and PDAC patients with Picro-Sirius Red (Fig. 8 A, B). The quantification of collagen deposits revealed an increase in area percentage in adipose tissues of S2-013 mPDAC than in that of their sham counterparts (Fig. 8C). We were also able to examine ECM deposition in visceral adipose tissue from PDAC patients. We quantified collagen deposition surrounding adipocytes and correlated the surge in pixel area percentage with increased circulating activin A levels (Fig. 8B, D). A significant increase in protein levels of fibronectin and collagen I/III was found within visceral adipose tissue from a patient with a greatly elevated level of circulating activin A in comparison to a patient with a relatively low level of circulating activin A, implying that adipose tissue could undergo remodeling in the cachectic state (Fig. 8E).

In addition, Picro-Sirius Red staining of both gonadal and subcutaneous adipose tissue obtained from C57BL/6 mice and cultured ex vivo with CM rich in activin A collected from KPC8069 cells revealed significant collagen deposition and adipocyte diameter reduction, similar to phenomena observed in vivo, which could be attenuated when supplemented with FST288 (Fig. 8F).

## Discussion

Pancreatic ductal adenocarcinoma (PDAC) is a notoriously lethal disease with indications that both adipose tissue and skeletal muscle wasting occurs early in disease progression^35^. Recent studies in mouse models have demonstrated a systemic increase of activin A, highlighting the atrophy of skeletal muscle cells^36^. Strikingly, current data from retrospective studies suggest no difference in survival between pancreatic cancer patients who lost skeletal muscle and adipose tissue mass and patients who lost only adipose tissue mass^10^. This challenges the current paradigm that muscle loss is the major complication in cancer cachexia and that cachectic progression is similar across different types of cancer. Our work provides new insight into the significance of visceral adipose tissue loss in PDAC and establishes the correlation between cachectic fat loss and autocrine/paracrine/endocrine signaling of activin A. We detected activin A expression in precursor PanIN lesions as well as PDAC tumor cells, which suggests that activin A from neoplastic cells predisposes PDAC patients to pre-cachexia status. We further revealed that activin A signaling is connected to the reduction of adipocyte size and the promotion of fibrosis in visceral adipose tissue from PDAC patients. The determinants of dramatic wasting of visceral adipose tissue in our patient population appear to transcend lipolysis and browning. Interestingly, the upregulation of SMAD and ERK signaling pathways in adipose tissue seem to indicate that activin A signaling mainly participates in visceral adipose tissue wasting. Although the metabolic crosstalk between the severity of PDAC and adipose tissue requires further investigation, our study demonstrates that activin A may be a key contributor among cancer-driven mediators for visceral adipose tissue loss in cachexia.

The first animal study regarding activin A and cancer cachexia was in the inhibin-deficient mouse model^37^. The deletion of inhibin elevated circulating activin A by 20-fold and manifested symptoms akin to human cancer cachexia^38^. A clinical trial in patients with cancer of the lung or the pancreas demonstrated that blockade of ACTRIIB using monoclonal antibody bimagrumab (BYM338) exerts an effect on preserving body weight and baseline of thigh muscle volume, total lean body mass, bone mineral density, and physical activity levels (NCT01433263). While BYM388 appears promising for recovery of skeletal muscle mass, there is no published clinical data regarding the effects of BYM338 on fat mass in patients with pancreatic cancer. To our knowledge, our study is the first to reveal the dose-dependent association between serum activin A levels and loss of visceral adipose tissue in PDAC patients.

The recently published report by Zhong et al. investigates human PDAC tumor expression of activins and related factors in relation to their effects on skeletal muscle loss. Our data concur with conclusions from their report regarding the choreography of systemic effects of activin A by PDAC tumors for the induction of cachectic pathogenesis in patients. The imbalance in activin A expression between PanIN lesions and tumor cells lends credence to a correlation between secretion of activin A and acceleration of cachexia in the disease state. The lack of change in activin A secretion between BxPC3 and PANC-1 cells suggests there may not be a relationship between *KRAS* or *p53* mutation status and the production of activin A for promotion of cancer growth (data not shown). Intriguingly, the maintenance of high levels of activin A secretion in KPC8069 cells after changing culture medium implies that there is continuous production of activin A from tumor cells with both *KRAS* and *p53* mutations.

Unlike phenomena observed in other studies, visceral WAT in PDAC patients and our mouse model displayed progressive loss of adipocyte function and size without undergoing the browning process. Our results instead demonstrate fibrotic remodeling of human and mouse visceral adipose tissue with elevated serum activin A, suggesting that activin A could induce trans-differentiation of white adipocytes into fibrotic cells. This is parallel to the implication of TGFβ signaling with fibrosis of subcutaneous fat in patients with cancer cachexia^25^. These shed new light on activation of SMAD-independent signaling pathways that has been described in non-adipose tissue^39–41^. Previous reports have posited that targeting of molecules downstream of activin A such as Twist1 or JNK ameliorates cancer-induced muscle cachexia and may prolong survival in animal models of PDAC^42,43^. In consideration of our findings, the activity of these molecules may also be targetable in adipose tissue. Given that we showed that browning of white adipose tissue is not the primary mechanism of cachectic fat loss in PDAC patients and mice, activin A seems to mediate an alternative mechanism for triglyceride degradation in visceral adipose tissue. Although our study design is limited to determine the effects of secreted activin A from PDAC tumor cells on visceral adipose tissue, further investigation is currently ongoing to examine the specificities of activin A signaling and the mechanisms by which activin A mediates cachectic fat degradation in PDAC. In summary, our study reveals a novel role of activin A in relation to the loss of visceral adipose tissue in the cachectic state in pancreatic cancer and may open new possibilities for the development of targeted therapies for the treatment of cachexia.

## Supplementary Information

Supplementary Information.

## References

[1] Argilés JM, Busquets S, Stemmler B, López-Soriano FJ (2014). Cancer cachexia: Understanding the molecular basis. Nat. Rev. Cancer.

[2] Barton MK (2017). Cancer cachexia awareness, diagnosis, and treatment are lacking among oncology providers. CA A Cancer J. Clin..

[3] Tisdale MJ (2002). Cachexia in cancer patients. Nat. Rev. Cancer.

[4] Fearon K (2011). Definition and classification of cancer cachexia: an international consensus. Lancet Oncol..

[5] Rahib L (2014). Projecting cancer incidence and deaths to 2030: the unexpected burden of thyroid, liver, and pancreas cancers in the United States. Can. Res..

[6] Henderson SE, Makhijani N, Mace TA (2018). Pancreatic cancer-induced cachexia and relevant mouse models. Pancreas.

[7] Tas F, Sen F, Keskin S, Kilic L, Yildiz I (2013). Prognostic factors in metastatic pancreatic cancer: Older patients are associated with reduced overall survival. Mol. Clin. Oncol..

[8] Baracos VE, Martin L, Korc M, Guttridge DC, Fearon KCH (2018). Cancer-associated cachexia. Nat. Rev. Dis. Prim..

[9] Wigmore SJ, Plester CE, Richardson RA, Fearon KCH (1997). Changes in nutritional status associated with unresectable pancreatic cancer. Br. J. Cancer.

[10] Kays JK (2018). Three cachexia phenotypes and the impact of fat-only loss on survival in FOLFIRINOX therapy for pancreatic cancer. J. Cachexia. Sarcopenia Muscle.

[11] Sah RP (2019). Phases of metabolic and soft tissue changes in months preceding a diagnosis of pancreatic ductal adenocarcinoma. Gastroenterology.

[12] Kir S (2014). Tumour-derived PTH-related protein triggers adipose tissue browning and cancer cachexia. Nature.

[13] Ohno H, Shinoda K, Spiegelman BM, Kajimura S (2012). PPARγ agonists Induce a white-to-brown fat conversion through stabilization of PRDM16 protein. Cell Metabol..

[14] Petruzzelli M (2014). A switch from white to brown fat increases energy expenditure in cancer-associated cachexia. Cell Metab..

[15] Argilés JM, Stemmler B, López-Soriano FJ, Busquets S (2015). Nonmuscle tissues contribution to cancer cachexia. Mediators Inflamm..

[16] Bloise E (2019). Activin A in mammalian physiology. Physiol. Rev..

[17] Qiu W, Kuo C-Y, Tian Y, Su GH (2021). Dual roles of the activin signaling pathway in pancreatic cancer. Biomedicines.

[18] Mancinelli G (2021). Role of stromal activin A in human pancreatic cancer and metastasis in mice. Sci. Rep..

[19] Zhao Y (2020). Oncogene-induced senescence limits the progression of pancreatic neoplasia through production of activin A. Can. Res..

[20] Loumaye A (2017). Circulating activin a predicts survival in cancer patients. J. Cachexia. Sarcopenia Muscle.

[21] Chen JL (2014). Elevated expression of activins promotes muscle wasting and cachexia. FASEB J..

[22] Narasimhan A (2020). Identification of potential serum protein biomarkers and pathways for pancreatic cancer cachexia using an aptamer-based discovery platform. Cancers.

[23] Han J, Meng Q, Shen L, Wu G (2018). Interleukin-6 induces fat loss in cancer cachexia by promoting white adipose tissue lipolysis and browning. Lipids Health Dis..

[24] Batista ML (2016). Cachexia-associated adipose tissue morphological rearrangement in gastrointestinal cancer patients. J. Cachexia. Sarcopenia Muscle.

[25] Alves MJ (2017). Adipose tissue fibrosis in human cancer cachexia: the role of TGFβ pathway. BMC Cancer.

[26] Batista ML (2013). Adipose tissue-derived factors as potential biomarkers in cachectic cancer patients. Cytokine.

[27] Sebastiano KMD (2013). Accelerated muscle and adipose tissue loss may predict survival in pancreatic cancer patients: The relationship with diabetes and anaemia. Br. J. Nutr..

[28] Lee JW, Komar CA, Bengsch F, Graham K, Beatty GL (2016). Genetically engineered mouse models of pancreatic cancer: The KPC model (LSL-Kras(G12D/+); LSL-Trp53(R172H/+); Pdx-1-Cre), its variants, and their application in immuno-oncology drug discovery. Curr. Protocols Pharmacol..

[29] Torres MP (2013). Novel pancreatic cancer cell lines derived from genetically engineered mouse models of spontaneous pancreatic adenocarcinoma: Applications in diagnosis and therapy. PLoS ONE.

[30] Taniguchi S, Iwamura T, Katsuki T (1992). Correlation between spontaneous metastatic potential and type I collagenolytic activity in a human pancreatic cancer cell line (SUIT-2) and sublines. Clin. Exp. Metas..

[31] Lee KM, Yasuda H, Hollingsworth MA, Ouellette MM (2005). Notch2-positive progenitors with the intrinsic ability to give rise to pancreatic ductal cells. Lab. Invest..

[32] Nguyen-Tu M-S (2019). Inflammation-linked adaptations in dermal microvascular reactivity accompany the development of obesity and type 2 diabetes. Int. J. Obes..

[33] Roa-Peña L (2019). Keratin 17 identifies the most lethal molecular subtype of pancreatic cancer. Sci. Rep..

[34] Hruban RH (2001). Pancreatic intraepithelial neoplasia: A new nomenclature and classification system for pancreatic duct lesions. Am. J. Surg. Pathol..

[35] Danai LV (2018). Altered exocrine function can drive adipose wasting in early pancreatic cancer. Nature.

[36] Zhong X (2019). The systemic activin response to pancreatic cancer: implications for effective cancer cachexia therapy. J. Cachexia. Sarcopenia Muscle.

[37] Matzuk MM (1994). Development of cancer cachexia-like syndrome and adrenal tumors in inhibin-deficient mice. Proc. Natl. Acad. Sci..

[38] Li Q (2007). Prevention of cachexia-like syndrome development and reduction of tumor progression in inhibin-deficient mice following administration of a chimeric activin receptor type II-murine Fc protein. Mol. Hum. Reprod..

[39] Bao YL (2005). Synergistic activity of activin A and basic fibroblast growth factor on tyrosine hydroxylase expression through Smad3 and ERK1/ERK2 MAPK signaling pathways. J. Endocrinol..

[40] de Guise C (2006). Activin inhibits the human pit-1 gene promoter through the p38 kinase pathway in a smad-independent manner. Endocrinology.

[41] Zhang L (2005). MEKK1 transduces activin signals in keratinocytes to induce actin stress fiber formation and migration. Mol. Cell. Biol..

[42] Mulder SE (2020). JNK signaling contributes to skeletal muscle wasting and protein turnover in pancreatic cancer cachexia. Cancer Lett..

[43] Parajuli P (2018). Twist1 activation in muscle progenitor cells causes muscle loss akin to cancer cachexia. Dev. Cell.

