# Supplementary material for: Visceral adipose tissue remodeling in pancreatic ductal adenocarcinoma cachexia: the role of activin A signaling
Source: Sci Rep. 2022 Jan 31;12:1659. doi: 10.1038/s41598-022-05660-7 (PMC8803848; doi:10.1038/s41598-022-05660-7)
Supplement: Supplementary file 1 — Supplementary Information. [file 41598_2022_5660_MOESM1_ESM.docx]

**Supplementary Material**

**Visceral adipose tissue remodeling in pancreatic ductal adenocarcinoma cachexia: the role of activin A signaling**

Pauline C. Xu^1#^, Mikyoung You^2#^, Seok-Yeong Yu^1^, Yi Luan^1^, Maya Eldani^1^, Thomas C. Caffrey^3^, Paul M. Grandgenett^3^, Kelly A. O’Connell^3^, Surendra K. Shukla^3^, Chandramohan Kattamuri^4^, Michael A. Hollingsworth^3^, Pankaj K. Singh^3^, Thomas B. Thompson^4^, Soonkyu Chung^2^*, So-Youn Kim^1^*

**Affiliations:**

^1^Olson Center for Women’s Health, Department of Obstetrics and Gynecology, College of Medicine, University of Nebraska Medical Center, Omaha, NE

^2^Department of Nutrition, School of Public Health and Health Sciences, University of Massachusetts Amherst, Amherst, MA

^3^Eppley Institute for Research in Cancer and Allied Diseases, University of Nebraska Medical Center, Omaha, NE

^4^Department of Molecular Genetics, Biochemistry, and Microbiology, College of Medicine, University of Cincinnati, Cincinnati, OH

# Both authors contributed equally to this work

* Co-corresponding authors

**Inventory of Supplementary Material**

**Supplementary Figures**

**Figure S1.**

**Figure S2.**

**Figure S3.**

**Supplementary Tables**

**Table S1.** List of primer sequences

**Table S2.** List of primary and secondary antibodies

**Supplementary References**

**Supplementary Fig. S1. Serum activin A increases in human Stage IV PDAC patients.** A. Additional immunofluorescence images of activin A (βA, symbol b in inset of hPDAC, AF488) and keratin 17 (CK17, symbol a in inset of hPDAC, AF568) expression in non-cancerous pancreatic tissue and tumor biopsies. Symbol c (yellow in inset of hPDAC) shows PDAC cells which have overlap of βA and CK17. Nuclei were stained with DAPI. Corresponding serum activin A levels are indicated in ng/ml. Scale bar = 50 µm. B. Pixel quantitation of DAPI, keratin 17, and activin A signals. C. Representative H&E images of pancreatic tissue from additional PDAC patients whose computerized tomography (CT) scans were included in Supplemental Fig. S3. Corresponding serum activin A levels are indicated in ng/ml. D. List of serum activin A and IL-6 levels in PDAC patients grouped by gender. E. Serum IL-6 levels in PDAC patients. F. Correlation between serum IL-6 and activin A. G and H. Serum activin A and serum IL-6 levels in PDAC patients grouped by gender. I. Correlation between activin A in sera and body mass index of PDAC patients. J. Correlation between activin A in sera and number of metastases sites of PDAC patients.


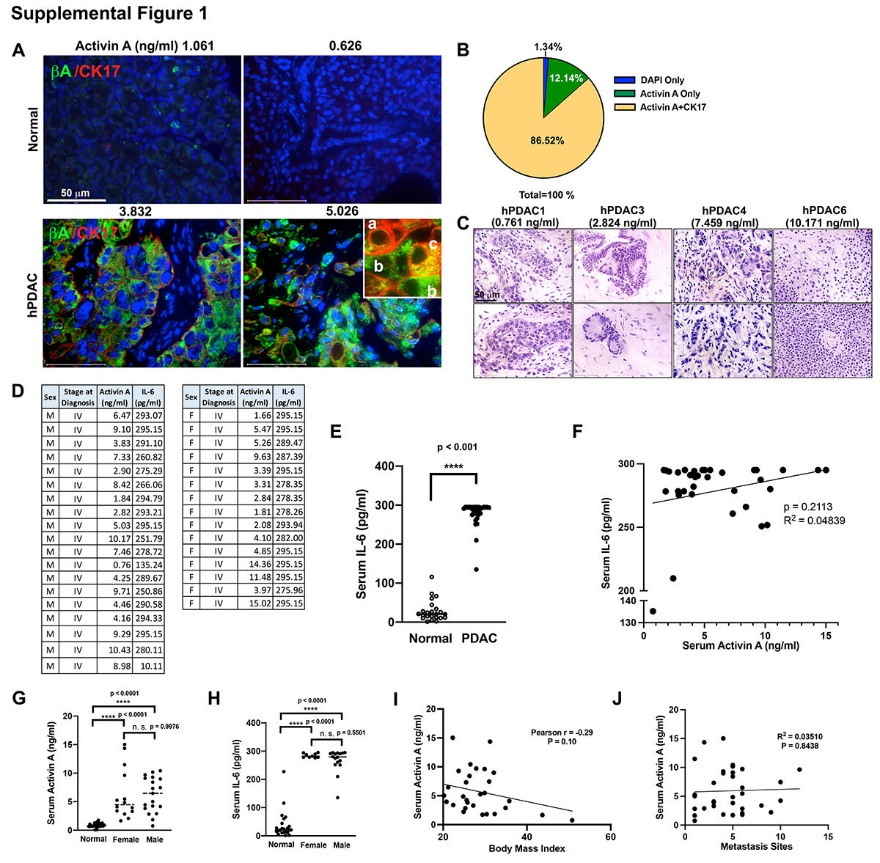


**Supplementary Fig. S2. Activin A is increased systemically in the disease state.** A. Correlation chart between activin A and IL-6 in sera of S2-013 mPDAC. B. Representative immunohistochemical images of activin A expression in tumor biopsies from additional PDAC patients whose visceral adipose tissue sections were analyzed in Figs. 2, 7, and 8. Corresponding serum activin A levels are indicated in ng/ml. C. Representative immunohistochemical images of activin A expression in draining lymph node, lung, spleen, and liver tissue sections from KPC 5127, 5921, 6065, and 6121 mice. Scale bar = 50 µm.

**
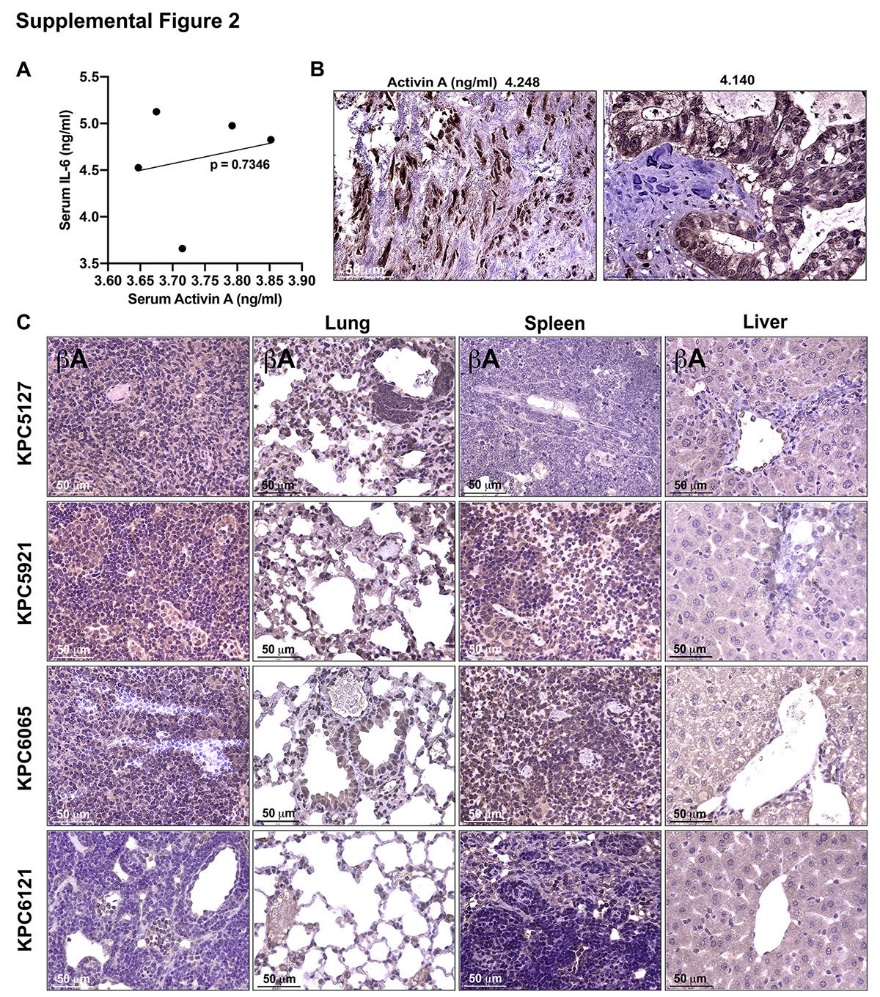
**

**Supplementary Fig. S3. Subctuaneous fat loss may be related to elevated activin A levels in human Stage IV PDAC patients.** Computerized tomography (CT) scans of Stage IV male PDAC patients at the third lumbar vertebrae. Corresponding serum activin A levels are indicated in ng/ml. Subcutaneous adipose tissue (blue) and visceral adipose tissue (yellow) are highlighted. Subcutaneous fat alone is shown in the bottom row.


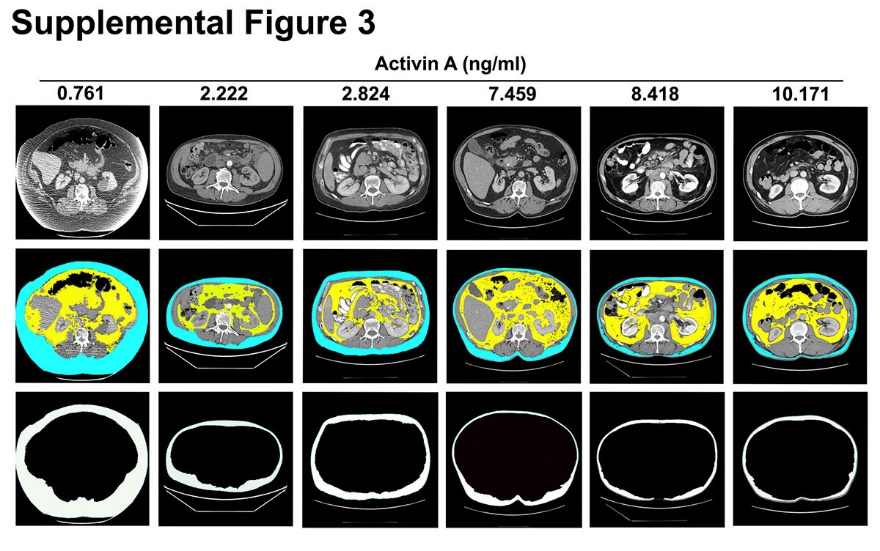


**Table S1.** List of primer sequences

| Gene | Primer sequence | Size | Species |
| --- | --- | --- | --- |
| *INHBA* | Hs01081598_m1 | 61 | Human |
| *Inhba* | Mm00434339_m1 | 65 | Mouse |
| *LIPE (HSL)* | Hs00193510_m1 | 67 | Human |
| *UCP1* | Hs01084772_m1 | 53 | Human |
| *RNA18S5*  *RNA45S5* | Hs03928985_g1 | 90 | Human |
| *Rn18S*  *Rn45S* | [Mm03928990_g1](https://www.thermofisher.com/taqman-gene-expression/product/Mm03928990_g1?CID=&ICID=&subtype=) | 61 | Mouse |
| *Rplp0 (36b4)* | F: 5’-GCTTCGTGTTCACCAAGGAGGA-3’  R: 5’-GTCCTAGACCAGTGTTCTGAGC-3’ | 135 | Mouse |
| *Adipoq* | F: 5’-GCACTGGCAAGTTCTACTGCAA-3’  R: 5’-GTAGGTGAAGAGAACGGCCTTGT-3’ | 122 | Mouse |
| *Lep* | F: 5’-TCTCCGAGACCTCCTCCATCT-3’  R: 5’-TTCCAGGACGCCATCCAG-3’ | 100 | Mouse |
| *Pparg* | F: 5’-GGCGATCTTGACAGGAAAGAC-3’  R: 5’-CCCTTGAAAAATTCGGATGG-3’ | 151 | Mouse |
| *Fas* | F: 5’-GGAGGTGGTGATAGCCGGTAT-3’  R: 5’-TGGGTAATCCATAGAGCCCAG-3’ | 140 | Mouse |

**Table S2.** List of primary antibodies

| **Antibody** | **Host** | **Dilution** | **Company** | **Catalog no.** |
| --- | --- | --- | --- | --- |
| Activin A | Rabbit | 1:1000 or 1:50 | Gifted by Dr. Wylie Vale | - |
| Cytokeratin 17 | Mouse | 1:50 | Santa Cruz Biotechnology | sc-393002 |
| Cytokeratin 19 | Mouse | 1:50 | Dako Omnis | GA615 |
| UCP1 | Rabbit | 1:1000 | Abcam | ab155117 |
| UCP1 | Rabbit | 1:50 | Abcam | ab10983 |
| p-Smad3 | Rabbit | 1:50 | Cell Signaling Technology | 9520 |
| p-Smad2/3 | Rabbit | 1:1000 | Cell Signaling Technology | 8828 |
| p-ERK | Rabbit | 1:1000 | Cell Signaling Technology | 4370 |
| p-p38 | Rabbit | 1:1000 or 1:50 | Cell Signaling Technology | 4511 |
| p-JNK | Mouse | 1:1000 | Cell Signaling Technology | 9255 |
| Fibronectin | Mouse | 1:50 | Novus Biologicals | MAB8258 |
| Collagen I alpha 1 | Mouse | 1:50 | R&D Systems | MAB6220 |
| β-actin | Mouse | 1:1000 | Sigma-Aldrich | A1978 |
| GAPDH | Mouse | 1:1000 | Santa Cruz Biotechnology | sc-137179 |

List of secondary antibodies

| **Antibody** | **Conjugation** | **Dilution** | **Company** | **Catalog no.** |
| --- | --- | --- | --- | --- |
| Anti-rabbit IgG | HRP | 1:2000 | Cell Signaling Technology | 7074 |
| Anti-mouse IgG | HRP | 1:2000 | Cell Signaling Technology | 7076 |
| Anti-rabbit IgG | Biotinylated | 1:200 or 1:400 | Vector Laboratories | BA-1000-1.5 |
| Anti-mouse IgG | Biotinylated | 1:200 or 1:400 | Vector Laboratories | BA-2000-1.5 |
| Anti-mouse IgG | Alexa Fluor 568 | 1:200 | Invitrogen | A-11004 |

**Supplementary References**

**[1-10]**

1. Jager-Wittenaar H, Dijkstra PU, Dijkstra G, Bijzet J, Langendijk JA, van der Laan BFAM, et al. High prevalence of cachexia in newly diagnosed head and neck cancer patients: An exploratory study. Nutrition. 2017;35:114-8.

2. Riccardi D, Allen K. Nutritional Management of Patients with Esophageal and Esophagogastric Junction Cancer:Several strategies can be incorporated to preserve or restore nutritional status of malnourished patients during management of esophageal cancer. Cancer Control. 1999;6(1):64-72.

3. Li H, Li Y, Liu Y, Huang D, Bai M, Ge S, et al. The incidence and impact of weight loss with cachexia in gastric cancer patients. Journal of Clinical Oncology. 2015;33(15_suppl):e20644-e.

4. Sun L, Quan XQ, Yu S. An Epidemiological Survey of Cachexia in Advanced Cancer Patients and Analysis on Its Diagnostic and Treatment Status. Nutrition and Cancer. 2015;67(7):1056-62.

5. Wilson HE, Stanton DA, Montgomery C, Infante AM, Taylor M, Hazard-Jenkins H, et al. Skeletal muscle reprogramming by breast cancer regardless of treatment history or tumor molecular subtype. npj Breast Cancer. 2020;6(1):18.

6. Sørensen J. Lung Cancer Cachexia: Can Molecular Understanding Guide Clinical Management? Integrative Cancer Therapies. 2018;17(3):1000-8.

7. van der Werf A, van Bokhorst QNE, de van der Schueren MAE, Verheul HMW, Langius JAE. Cancer Cachexia: Identification by Clinical Assessment versus International Consensus Criteria in Patients with Metastatic Colorectal Cancer. Nutrition and Cancer. 2018;70(8):1322-9.

8. Paajanen J, Ilonen I, Lauri H, Järvinen T, Sutinen E, Ollila H, et al. Elevated Circulating Activin A Levels in Patients With Malignant Pleural Mesothelioma Are Related to Cancer Cachexia and Reduced Response to Platinum-based Chemotherapy. Clinical Lung Cancer. 2020;21(3):e142-e50.

9. Rimar KJ, Glaser AP, Kundu S, Schaeffer EM, Meeks J, Psutka SP. Changes in Lean Muscle Mass Associated with Neoadjuvant Platinum-Based Chemotherapy in Patients with Muscle Invasive Bladder Cancer. Bladder Cancer. 2018;4:411-8.

10. Kim HL, Han KR, Zisman A, Figlin RA, Belldegrun AS. Cachexia-like, symptoms predict a worse prognosis in localized T1 renal cell carcinoma. Journal of Urology. 2004;171(5):1810-3.
